# Commissioning, Implementation and Policy Perspectives on Interpreting Services in UK Primary Care: A Qualitative Study

**DOI:** 10.34172/ijhpm.9181

**Published:** 2026-07-13

**Authors:** Judith Yargawa, Katriina L. Whitaker, Graham Hieke, Cecilia Vindrola-Padros, Emily D. Williams, Paramjit Gill, Lily Islam, Sabine Braun, Georgia B. Black

**Affiliations:** ^1^Wolfson Institute of Population Health, Queen Mary University of London, London, UK.; ^2^School of Health Sciences, University of Surrey, Guildford, UK.; ^3^Department of Targeted Intervention, University College London, London, UK.; ^4^Faculty of Life Sciences and Medicine, King’s College London, London, UK.; ^5^Warwick Applied Health, Warwick Medical School, University of Warwick, Warwick, UK.; ^6^Patient and Public Involvement, London, UK.; ^7^Centre for Translation Studies, University of Surrey, Guildford, UK.

**Keywords:** Interpreting, Language Support, Primary Care, Policy, Qualitative Research, United Kingdom

## Abstract

**Background:**

Language barriers in primary care contribute to health inequalities, limiting access to services and affecting patient outcomes. Professional interpreting services are emphasised in UK guidance, but evidence on how policy-makers and commissioners deliver these services is lacking. This study aimed to explore the commissioning and implementation of interpreting services in UK primary care from the perspectives of commissioners, policy-makers, interpreters, and interpreting service providers.

**Methods:**

Semi-structured interviews were conducted with 31 participants (12 national policy-makers, 6 commissioners, 7 interpreters, and 6 interpreting service providers). Thematic analysis was conducted to explore delivery and identify best practices and key challenges in delivery.

**Results:**

The study found variation in commissioning models, with some areas favouring large national providers for widened scope and cost efficiency, while others prioritised local interpreting services for higher quality. The UK was seen as world-leading in interpreting provision, however fragmentation, lack of standardisation, accountability gaps, funding constraints within a publicly-funded healthcare system, and varied interpreter competencies and renumeration led to inconsistency in delivery. The healthcare sector was reported as having lower interpreting standards compared with other UK public sectors. Technological solutions were used in delivery and offered key advantages but sometimes failed to meet patient and provider needs.

**Conclusion:**

Strengthening national regulation, funding allocation, and service integration is essential to addressing systemic issues and improving interpreting service delivery in UK primary care. Policy recommendations include promoting "what good looks like," standardising interpreter qualifications and adopting approaches tailored to local population needs.

## Background

**Key Messages**

**Implications for policy makers**

- Implementation of interpreting services involves multiple stakeholder groups including public and private sector actors. Interconnectedness and robust feedback mechanisms between these stakeholders enable nuanced understanding of the factors influencing service delivery.
- Fragmentation, lack of standardisation, funding, and varied interpreter competencies impede quality delivery. Addressing these policy and structural issues are crucial to improving interpreting service delivery.
- Tailoring interpreting services to the local context is important, but standardisation is needed; “what good looks like” requires promotion across institutions.
- A multifaceted approach is needed in addressing identified challenges, which will involve potentially immediate fixable solutions to addressing wider structural issues including fragmentation within healthcare, funding, the rapidly evolving artificial intelligence (AI)/digital tool landscape, and the diminishing pool of interpreters.

**Implications for the public**

Interpreting services are key to bridging language barriers between patients and healthcare professionals. Policy-makers, commissioners, interpreting service providers and interpreters play important roles in delivering interpreting services to patients and it is key to understand factors that enable or impede their delivery of these services. Use of different standards, funding challenges and interpreters having varying levels of skills affect interpreting delivery. To improve delivery, consistent standards need to be used, funding allocated appropriately and what “good looks like” promoted. Stakeholders who deliver services need to also work together and provide cross-feedback to ensure provision of quality services.

The United Kingdom is a linguistically diverse nation, with a substantial proportion of the population speaking languages other than English.^1,2^ This necessitates the provision of interpreting services within primary care to ensure equitable healthcare access.^3-6^ Accurate and accessible communication is fundamental to effective healthcare delivery, enabling accurate diagnosis, informed consent, and patient understanding of treatment plans.^7^ Without it, disparities in healthcare access and outcomes are inevitable.^4,8,9^ Several policy frameworks mandate the use of professional interpreters in healthcare settings, most notably the Equality Act 2010, which prohibits discrimination on the grounds of language.^10^ The National Health Service (NHS) England policies further reinforce this commitment, emphasising the importance of professional interpreting for effective communication and patient safety.^4,11^ However, despite these policy directives, there are concerns about implementation of interpreting services in primary care, particularly concerning access, effectiveness, and potential unintended consequences.^8^

 Current UK policy strongly favours the use of formal, qualified interpreters over reliance on family members or friends.^11^ This preference stems from concerns about accuracy, confidentiality, and potential power imbalances within family dynamics. Furthermore, tensions arise between safeguarding, confidentiality, and patient autonomy in interpreting choices.^12^ The role of private companies in interpreting service provision also raises important questions regarding quality assurance, regulation, and cost-effectiveness.^13^ The fragmented nature of the market can lead to inconsistencies in service quality and potential challenges in ensuring interpreter competency. In addition, whilst codes of conduct for professional interpreting exist,^14,15^ what “a good service looks like” may not always be known or readily implementable in healthcare.

 The cost of interpreting services is significant. Public sector organisations are advised to work with language service providers who vet and manage freelance interpreters, ensuring they meet the necessary qualifications and experience.^11^ These providers also employ technological solutions to streamline the booking process and monitor demand. However, industry commentators suggest that procurement should not solely focus on cost; the quality of service must be balanced with pricing considerations to avoid issues arising from underqualified interpreters, especially in “high-stakes” fields like healthcare.^13^

 While the policy framework for interpreting in primary care is relatively well-established (See Table 1), limited research explores how these policies are translated into practice.^16^ Existing studies often focus on the perspectives of healthcare professionals, with less attention given to the experiences of interpreters themselves, policy-makers, and commissioners. This study aimed to address these gaps by exploring the relationship between policy, policy implementation, and delivery of professional interpreting services in primary care. Specifically, the study aimed to explore the commissioning and implementation of interpreting services in UK primary care from the perspectives of commissioners, policy-makers, interpreters, and interpreting service providers.

**Table 1:** Key National Policy Frameworks for Interpreting in UK Primary Care

**Law/Guideline**	**Stipulations**
Equality Act 2010^10^	Outlines the duty to make reasonable adjustments and the prohibition of discrimination. Outlines the Public Sector Equality Duty for the NHS and other public services to take proactive steps to advance equality of opportunity including language accessibility.
The NHS Constitution for England^17^	Stipulates that NHS organisations should aim to “*contribute towards providing fair and equitable services for all and play your part, wherever possible, in helping to reduce inequalities in experience, access or outcomes between differing groups or sections of society requiring health care.*” Not enforceable but has legal backing through laws such as the Equality Act 2010.
Health and Social Care Act 2012^18^	NHS bodies have a statutory duty to “*have regard to*” the constitution when making decisions. Not enforceable but has legal backing through laws such as the Equality Act 2010.
NHS England Guidance for Commissioners: Interpreting and Translation Services in Primary Care (published 2018)^11^	Published guidance to support commissioning of interpreting in primary care, published by the NHS England Primary Care Commissioning Team. States that commissioners must conduct population needs assessments to determine demand for interpreting services, considering factors like language diversity and migration patterns. States that ICBs are responsible for ensuring interpreting services are accurate, timely and professional, and ensure that providers comply with both contractual and professional standards.

Abbreviations: NHS, National Health Service; ICBs, Integrated Care Boards.

## Methods

###  Theoretical Framework

 Our study was underpinned by the Consolidated Framework for Implementation Research,^19,20^ with its five constructs used to guide our data collection and structure the data analysis: *Intervention characteristics* (delivery and quality of interpreting services); *outer setting* (local policy, national policies, guidance, commissioning, and contracting procedures); *inner setting* (technology, interpreting service provider characteristics, operational and organisational structure); *characteristics of individuals* (training on interpreting services, experiences and knowledge of interpreting services); and *process *(planning of interpreting services, operations, and tasks).

###  Study Design, Sampling, and Recruitment

 This paper presents qualitative data from a wider project, the INTERPRET-X Study (NIHR134866). We explored the implementation of professional interpreting in primary care through interviews with key stakeholders and primary care sites across England representing diverse population and practice characteristics.

 Purposive sampling was used to include a range of professional backgrounds to understand diverse experiences of interpreting service delivery and obtain in-depth, holistic information.^21^ We selected 31 participants from a range of experiences and perspectives related to interpreting services in primary care (Supplementary file 1). This included Integrated Care Board (ICB) commissioners commissioners (6); national and local interpreting service providers (6); interpreters (7); and national policy-makers (12, particularly, from different healthcare civil service organisations (7), national think tanks and charities (2), and interpreting regulator and professional interpreting/translation associations (3)). Participants were identified through a prior case study which we conducted in primary care sites,^22^ the study’s Steering Group and Advisory Group, our networks and snowball sampling. Participants were approached online and given information sheets, with any further questions answered via email or Microsoft Teams call. Written informed consent forms were shared and participants who consented took part in the study.

###  Data Collection

 Semi-structured interviews were conducted with participants remotely by the first author (experienced qualitative researcher, PhD, female). We also observed interpreting delivery in primary care and interviewed primary care staff, which have been reported elsewhere.^22^ Interviews explored participants’ experiences with interpreting services including: the current state of interpreting in the country, quality of interpreting services, barriers and enablers to usage and implementation, funding models and resource implications, recruitment of interpreters, requirements for providers, local population and patient needs, and engagement with interpreting services. The research objectives and a pilot study that we conducted^1^ guided development of the topics explored. Whilst there were cross-cutting questions for the different stakeholder types, questions were generally tailored to each specific stakeholder group, hence separate topic guides were used for each stakeholder type (Supplementary file 2). All interviews were conducted remotely via Microsoft Teams and audio- or video-recorded with consent. The sessions were then transcribed verbatim by a certified third-party professional transcription company. The interviews lasted on average 45 minutes to an hour and were undertaken between February and May 2024.

###  Data Analysis

 Data were analysed thematically using Rapid Research and Evaluation Lab Rapid Assessment Procedure (RAP) sheets^23^ and Microsoft Office packages. Detailed notes and summaries were made for each participant category (eg, commissioners, national stakeholders) and categorised under themes and sub-themes. This method of analysis facilitated comparisons across our various study sites and stakeholder groups, and also data triangulation. Data analysis followed an iterative process, with interview transcripts and notes reviewed with data collection, identifying gaps and areas for in-depth analysis. RAP sheets and notes supported preliminary analysis and guided group discussions among the research team (JY, GBB, CV, KW, GH). This approach enabled the identification of good practice examples and areas for improvement, which informed the study’s recommendations.

## Results

 Our findings are organised in two sections: the first, reporting how participants described the way interpreting services are designed (commissioned and funded); the second, detailing the way interorganisational relationships and other structural factors affect delivery.

###  Design of Interpreting Services for Primary Care

####  Commissioning of Interpreting Services

 Interpreting services were commissioned through ICBs, who contracted providers according to local population needs. Commissioning models ranged from centralised frameworks covering entire regions and ICBs to decentralised systems where individual or a group of boroughs or general practices (GPs) arranged their own provision. Commissioning contracts differed across boards. Commissioners from one area described having one main national provider for all its primary care practices, with some back-up options. In contrast, commissioners from a different area described a strong preference for having local providers because they were more attuned to local population needs and provided high quality. They developed strong working relationships over time and were preferred despite considerable increased cost compared to a national (telephone-based) provider. Participants from one area with relatively high interpreting need described total devolvement to local (borough-level) contracts, with several primary care practices or boroughs having multiple providers and hiring their own interpreters or health advocates (health advocates are frontline staff who interpret, but have received further training to also provide basic health information to patients and can speak on their behalf during consultation).

 Key performance indicators (KPIs) were used to assess interpreting delivery (See Box 1). Some participants described assurance methods such as portals with feedback from primary care practices (eg, positive comments, complaints, complaint resolution), periodic surveys with practices on service performance, and contract monitoring meetings/reviews with interpreting providers.

**Box 1. Examples of Key Performance Indicators for Interpreting Service Providers**

- Coverage of core general practice hours (8:00 am to 8:00 pm hours)
- Being available within a reasonable time (eg, for telephone, percentage of patient calls answered within a minute)
- Reengagement following call cut-offs (eg, <1 minute)
- Minimum interpreter qualifications
- Patient satisfaction and experience
- Fulfilment/requirements regarding rare languages (ability to provide interpreting services for rare languages)
- The percentage of bookings that are met and unmet (and future mitigation plans for unmet bookings)
- Service and activity monitoring (eg, complaints made and resolutions, number of calls, number of face-to-face sessions)

####  Funding of Interpreting Services

 Participants reported that ICBs generally adopted a pay-per-activity model, where costs were calculated based on factors such as the number of patients and the duration of interpreting services. Telephone interpreting was typically charged per minute, whereas face-to-face interpreting was sometimes billed based on a minimum duration, such as half-an-hour, although some respondents noted that this could vary. It was highlighted that face-to-face interpreting was more expensive than telephone services and often included travel costs. Some ICBs addressed the challenges of providing services in rural areas by incorporating travel mitigation strategies in their KPIs such as reimbursing travel expenses, prioritising interpreters who were able to drive, and offering remote interpreting options. Where ICBs did not cover travel expenses, interpreting service providers had to meet these, resulting in reliance on local interpreters to minimise travel costs.

###  Policy and Structural Factors Impacting Interpreting Delivery 

 Delivery of interpreting services in healthcare was driven by a range of policy and system factors, inter-organisational relationships, as well as interpreting provider markets, interpreter competencies and primary care infrastructure (See Figure).

**Figure:**
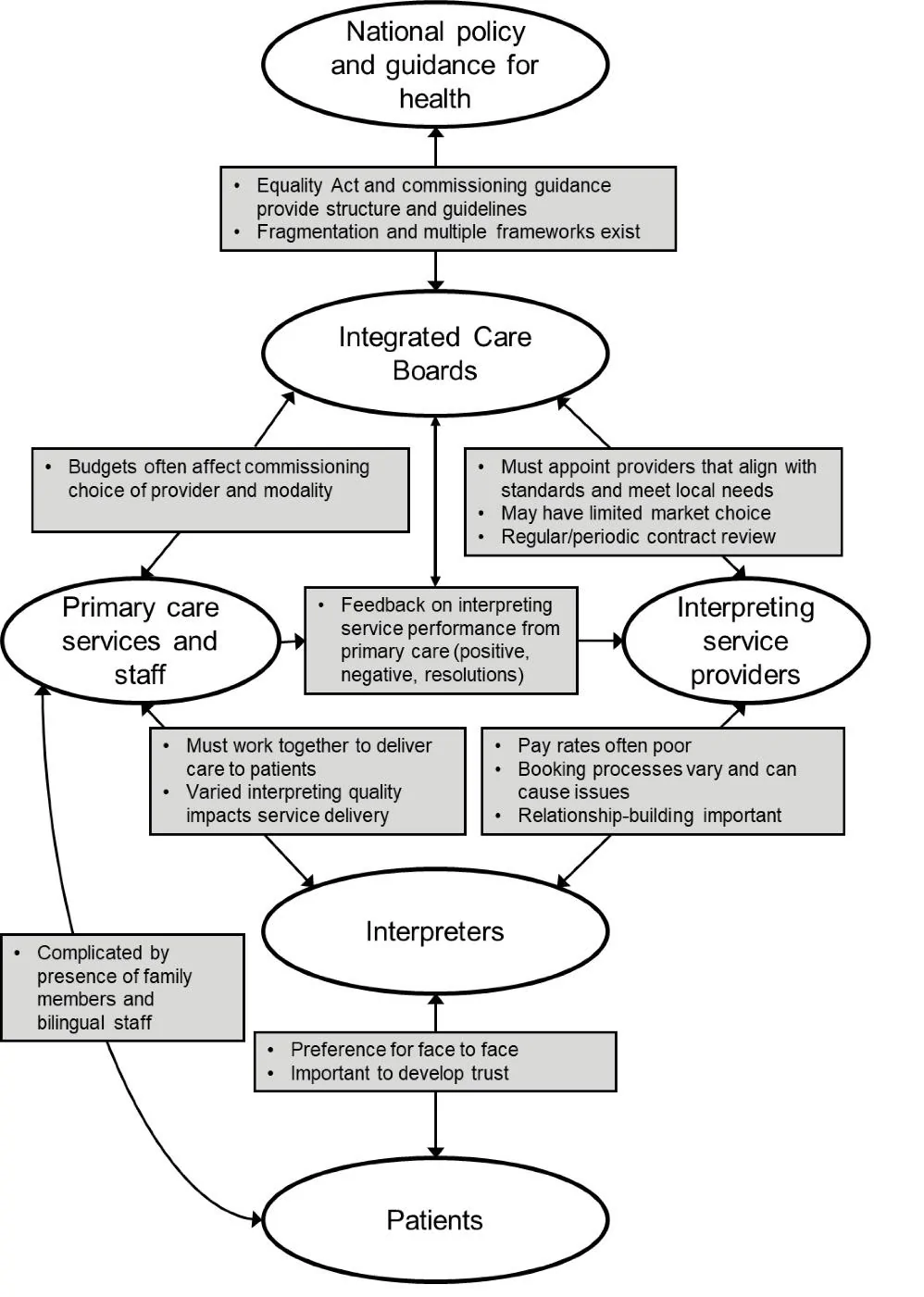
Interdependency of Health Stakeholders in Interpreting for Primary Care Delivery.

####  NHS Fragmentation and Lower Interpreting Requirements Compared to Other UK Public Sectors

 UK interpreting provision was seen by a few national stakeholders as world-leading compared with other European countries and robust, albeit challenged by NHS fragmentation. The health sector was seen as a particularly challenging environment for interpreting provision compared with other public bodies. National stakeholders highlighted the usage of different frameworks for commissioning interpreting services in the NHS, with no “*consistent approach to setting standards.” *To address this, it was reported that one ICB is working towards having a single, unified commissioning framework to improve consistency and equity of access. Healthcare interpreting delivery was also sometimes portrayed as a “poor cousin” in relation to policing or the justice system. Participants felt that NHS interpreting standards had declined over the past decade due to low pay rates for interpreters, contracting arrangements and the predominance of large national framework contracts procured through interpreting service providers. There was a view particularly amongst participants working at a national policy level that interpreting required better standardisation and regulation.

 “*There are about seven or eight different contracts [in the NHS] …There’s loads of different frameworks that the- There’s no standards. Each trust- It is the Wild West... It’s the Wild West in the NHS. Not so in the police. It’s very structured …. The NHS has to get a grip of this on a national basis” *(HS2, National stakeholder).

####  Policy Gaps, Apathy and Lack of Accountability 

 Despite existence of published guidance to support commissioning of interpreting in primary care, participants reported that compliance was not monitored, and that the “*policy can be very separate from what’s happening on ground.”* One national stakeholder reported that an ICB lead had told him that it was not their responsibility to appoint interpreting services despite the clear guidance. More broadly, it was not clear who was accountable for service provision and delivery either nationally or locally. Some stakeholders mentioned that it would be beneficial to know what a good service looked like, since many different interpreting and accreditation professional organisations exist, *“but there isn’t a kind of definitive body or set of standards which are all agreed to.*”

 National policies on interpreting were seen as lacking in political will and leadership, although a lead for language services was appointed recently which was seen as a step in the right direction. Some participants suggested that this could be related to structural racism, or at least, apathy towards people with language needs, with another participant highlighting the intersectionality of race with language needs. Some participants felt there was also a lack of appreciation in national government and local authorities for the value of interpreting and bilingualism more widely, and that this was restricting the pipeline of trained interpreters. A national stakeholder revealed that large parts of the UK lacked access to interpreting services, making it difficult for these areas to engage with the market, and interpreting services were often not prioritised.

####  Challenges in Funding and Contracting 

 Funding for interpreting services in the NHS posed significant challenges, with uncertainty about who was responsible for financing and holding liability. There was no specific budget allocated for interpreting services; instead, participants reported that funding had to be sourced from budgets intended for general GP services. Participants discussed the financial pressures facing ICBs, with the government imposing efficiency savings of up to 30%. One ICB respondent mentioned that interpreting services were sometimes perceived as an avoidable cost, with some questioning why people could not speak English. Interpreting service providers and commissioners often had a collaborative but sometimes complex relationship (See Figure).

 A key issue was choice. There was limited pool of providers in some areas able to meet the standards set in tenders, with competition narrowed to just one or two successful applicants. While there had been opportunities to engage more providers outside the NHS Framework for contracting, some participants felt that this approach could compromise service quality and patient care. Other participants felt that the KPIs were too basic, limiting the ability to hold providers accountable. One commissioner described the practice of resorting to conducting mini-competitions, aiming to secure local providers and bring greater rigour to contract management.

*“It’s a very large framework, it’s got multiple providers for all of the different interpreting and translation needs. But what we do find with the framework is that there’s no contract management in place, … and there’s not very much opportunity to hold providers to account, so very basic KPIs that aren’t really monitored as rigorously as we would like them to be … So what we then do is when they re-procure that framework, we then re-procure a mini competition, which is essentially we can use any of the providers that are on that framework, but we go through our own procurement, so we ask questions, we invite people to bid, and submit their own bids, and then what we say is that we will work with those providers closely, so they will get our activity, they will get our bookings” *(GC3, Commissioner).

####  Challenges in Regulation, Market Dynamics and Quality of Interpreting Services

 Interpreting service providers differed in size, scope and operations. There were trade-offs in choosing an interpreting service provider, with implications for delivery (Table 2). A national stakeholder highlighted that the language services market was relatively robust and continuously growing, particularly in terms of its capacity to meet service bookings. However, there were also concerns about its behaviour regarding meeting NHS needs vs. leveraging its commercial position. Overall, the lack of regulation within the interpreting industry concerned participants, with some providers calling for a government body to oversee the sector. This lack of oversight meant that anyone could set up an interpreting service and treat interpreters as they saw fit, which raised concerns about fair pay and professional standards. Following incidents that highlighted inadequacies (for example, being distracted whilst interpreting), one local ICB responded by establishing minimum qualification standards for freelancers and removing under-performing interpreters from contracts.

**Table 2:** Large Versus Small Interpreting Service Providers (Gleaned and Inferred From Respondent Reports)

**Description**	**Small Interpreting Service Providers**	**Large Interpreting Service Providers**
Operations	Strong local presence Locally developed for community Focused on local needs and trust-building	Cover large and multiple geographical areas Prioritise availability and delivery of on-demand services
Interpreting modality	Primarily face-to-face interpreting	Primarily telephone interpreting, but also deliver all modalities including face-to-face
Key strengths	The “personal touch” - tailored services that address need for “cultural interpreting” and social prescribing Foster continuity and a sense of belonging within local communities Face-to-face provision seen as particularly high quality	On-demand services that maximise technology Ease of access through efficient platforms and speedy services Delivery at scale Telephone interpreting seen as cost-effective and flexible
Key weaknesses	More expensive than larger providers Less reliable, inconsistent supply due to a lack of volume Some instability and diminished capacity to meet demand	Lack deep connections with local areas Described as “faceless” Seen as profit-driven
Relationship-building with interpreters	Seen as important More likely to encourage interpreters to pursue qualifications, sometimes offering financial incentives Facilitate meetings between commissioners and interpreters, providing a platform to discuss challenges	Seen as important Some recognition incentives in place, eg, for an interpreter reaching their 100^th^ appointment of the year Resolve incidents and support interpreters following challenging situations (eg, violent or other distressing events during work)
Mechanisms for relationship-building with interpreters	Coffee meetings and get-togethers to gather feedback Recognition mechanisms Annual general meetings to gather feedback Self-referral options for patients to contact interpreting services directly	
Relationships with other providers	Despite competition, small providers sometimes worked with and supported large providers that had won contracts that the small providers had lost	

 “*…Anybody could set up an interpreting service, just anybody and they could treat their interpreters how they like... It needs some sort of guidance, you know, make sure that everybody has got a qualification, that everybody has got the checks. That people are paid and treated well. It just needs a little bit more, it needs a government body really to support it” *(FM5, Interpreting service provider).

 “*… The current marketplace is just a disorganisation of … [interpreting] companies appointing interpreters and organisations trying to buy interpreters” *(HS3, National stakeholder).

####  Interpreter Payment Structures, Recruitment and Retention Challenges

 Participants reported that prices for interpreting services were determined by government and public service procurement processes, which set the price paid to interpreting service providers. However, these contracts did not specify what service providers should pay their interpreters. A large interpreting service provider mentioned that “*we will pay higher rates to more qualified people [interpreters]. So generally, the more qualified you are, the more attractive or higher-paid work that you can get*….” Pay rates for interpreters had remained stagnant in recent years or dropped, sometimes below official minimum rates. One interpreter, for example, had seen their pay drop from £32 to £16 per hour, prompting them to leave the sector for private work where the pay was better. Other public and private sector organisations such as the police, legal firms, and insurance companies offered significantly better pay. Some sectors also provided block contracts for interpreters, offering consistent work for set hours each week. Policies around travel were also inconsistent, with some agencies covering travel costs, not covering, or only covering transport costs if requested before the appointment. Interpreters were typically paid less for telephone interpreting compared to face-to-face services. For telephone assignments, interpreters were paid only for the call duration, with no compensation for waiting time or availability on the app. This contrasted with face-to-face work, where interpreters were paid for the full session.

 “*It’s not worth it at all like 20 minutes talk you’ll get £5.00 so I don’t feel like it’s worth it. …It’s the rate for telephone. Like per minute … 26p for per minute so it’s not okay so I don’t take telephones … Face to face at least like minimum one hour they have to pay. If you go over one hour every minute they will pay like I think 50p. So basically it’s local like where I live if it’s local patient they will pay £16.00 an hour and £20.00 for travel cost and if I go different borough like [name of borough] they will pay £16.00 same rate but £8.00 for the travel cost” *(EI4, Interpreter).

 Interpreters also highlighted competition that could arise from booking processes: some providers used online portals with a first-come, first-served system, whereas others relied on email-based job dispatch. Participants noted that the competitive nature of the booking process often meant that interpreters needed to quickly choose from a pool of available options, which could affect job opportunities. Small interpreting companies also reported financial constraints as a major issue and losing interpreters to other job roles, such as factories where pay was more attractive. These companies, particularly post-COVID-19, found it challenging to recruit interpreters for face-to-face work due to reduced public sector funding.

 Some national stakeholders expressed concern over the diminishing pool of interpreters, citing factors such as pay, terms and conditions, and the overall attitudes towards the profession as key contributors to this trend. The sector was seen as increasingly unattractive to potential interpreters. Additionally, Brexit and the fact that languages were no longer being taught widely in schools were also highlighted as contributing factors to the shrinking pool of qualified interpreters. Interpreting also lacked sufficient recognition as a skilled profession, further driving professionals away from the sector. A number of respondents highlighted the need for better recognition of the value of multi-lingualism and capturing interpreting skills within the community. Stakeholders noted that training new interpreters would take considerable time and resources, which needed to be factored into any long-term planning. Some stakeholders brought up English-speaking competency within the population. Several respondents across the various groups interviewed emphasised the need for non-English speakers to learn English, for example, for better self-expression in mental health consultations.

####  Awareness, Technology and Infrastructure Issues Within General Practices

 Participants identified several barriers to the delivery of interpreting in primary care. One of the main issues was the lack of awareness among frontline staff about how interpreting services function. One national stakeholder noted that healthcare providers often claimed to have interpreting services like Language Line available but had limited knowledge of how to use them effectively. Participants indicated that interpreting needs in other primary care sectors, such as pharmacies, might be under-recognised.

 Issues with technological infrastructure were particularly noted by interpreting service providers, such as interpreters using low-cost phone networks or poor wi-fi connectivity, outdated equipment, rurality and old buildings in healthcare settings. GP surgeries were often more open to telephone interpreting due to its on-demand nature, but attitudes towards technology were mixed. Some NHS organisations were hesitant to adopt technology used in other public sectors, because despite encryption and safety measures, “*we’ve got to vet your app*.”

 “*… If the interpreter is … on … one of the cheaper networks, … those types of networks don’t provide you with the very bests of coverage. So an interpreter can easily drop off a call …. And it’s the same really with video interpreting. You’re at the mercy of wi-fi and the number of hospitals I’ve gone into four, five years ago where their wi-fi, very, very thick walls in old school hospitals and some GP practices. The wi-fi isn’t up to scratch to be able to go and do what we’re doing now. It’s improving, it’s got to improve. But you will still walk into a number of NHS Trusts and hospitals around, especially [names area], and they aren’t where they should be in terms of their wi-fi to be able to support video interpreting” *(FM1, Interpreting service provider).

 Interpreting service providers described technological solutions that they introduced to improve service delivery and address some of these challenges, such as the “re-join” feature which allowed GPs to reconnect to the same interpreter if the call line dropped, and providing free tablets to health facilities to encourage use. NHS England has also introduced Card Medic, a smart app with basic phrases in multiple languages which healthcare professionals can use to overcome language barriers.

 “*Some ICBs, they – Integrated Care Boards – they are very mature in their adoption of technology, and they’re very comfortable using an app for telephone or for video interpreting. And then at the other end of the scale, we get some hospital trusts or ICBs that won’t use technology at all. And then the bulk is somewhere in the middle” *(FM3, Interpreting service provider).

## Discussion

 This study investigated the design and delivery of professional interpreting services in UK primary care, focusing on the various stakeholders involved in service provision. The study examined the roles of key bodies involved in the provision of interpreting services, and how policy and structural factors collectively shaped the delivery of interpreting services in primary care settings.

 Our findings demonstrate the interdependencies between national policy-makers, ICBs, interpreting service providers and interpreters. These four bodies were connected by key top-down principles of equity and access, pay rates and KPIs. Their ability to collectively implement policy and improve delivery of interpreting services was hampered by fragmentation, varied standards, financial pressures and dwindling interpreter numbers.

###  Interpretation, Implications, and Recommendations

 The study found variation in how interpreting services are commissioned and delivered in UK primary care in line with previous studies in this area.^7,8^ While some areas prioritised local, community-based interpreting providers for higher service quality, others relied on large national providers for cost efficiency. Similarly, a realist review by Jager et al found that commissioners sometimes chose to make funding decisions based on little evidence of service quality or delivery because they felt pressure to prioritise financial issues.^24,25^ This is further undermined by persistent organisational fragmentation in integrated care models.

 Fragmentation and lack of standardisation within the health sector emerged as key issues impacting delivery, with the NHS seen as having multiple interpreting frameworks and lower interpreting standards compared with other UK public sectors. It may not be possible to statistically evidence higher interpreting quality in one sector over another as these data are not readily available. The perception that interpreting standards are higher in the justice sector compared with the health sector may have arisen from policies developed for the criminal justice system: a national agreement which ensured, while it was still in place, that qualified and registered interpreters are used,^26,27^ and a European Union directive which established the rights of accused/suspected persons with limited language skills to interpretation and written translation.^28^ In addition, the justice system typically requires Level 6 qualification (Diploma in Public Service Interpreting) compared with the health sector’s Level 3 requirement (Certificate in Community Interpreting), which may have contributed to perceptions of differential interpreting quality. However, there have also been reports of inconsistencies in interpreting provision and quality in the justice sector due to private sector outsourcing.^29^ In spite of these, the health sector might still glean some lessons from these other UK public sectors, particularly relating to standardisation. While a national framework may not be feasible in the health sector since interpreting services have to be tailored to the local context, some standardisation might be possible across cross-cutting issues and “what good looks like” could be promoted across institutions. Where possible, the same frameworks could be used at regional or ICB-level. The plan towards adopting a single, unified framework in one ICB is welcome news and could be replicated in other areas. The NHS could also learn from the Police Approved Interpreters & Translators scheme, which has increased interpreting standards and interpreter working conditions.^30^ Cross-governmental guidance, as suggested by a respondent, may serve as a channel for fostering a shared learning environment.

 Funding also impacted interpreting delivery. There may be funding constraints in a publicly funded healthcare system due to competing priorities and resource allocation issues. Resources are generally finite and with the health sector already overstretched resource-wise, providing additional funding may not be a clear-cut solution. However, there might still be scope to improve funding allocation so that resources are directed where needs are greater.^31^ A national stakeholder suggested that the NHS might need to be pragmatic and make reasonable compromises in interpreting delivery, as used in the asylum system in certain European countries. Some of these countries use remote interpreting, for instance for rare languages/dialects in order to improve access to interpreters and the speed of interpreting provision,^32^ although it may reduce interpreting quality compared to on-site interpreting.

 Interpreting delivery depends on interpreters directly, hence efforts should be made to improve their pay rates and working conditions. Additional pipelines for increasing the pool of interpreters should be explored, such as language training and harnessing the “low hanging fruit” of people already fluent in communities. Varied interpreter competencies emerged as an issue impacting delivery. Codes of conduct for interpreters exist, published by recognised professional interpreter and translator associations and describing what “a good service looks like.”^14,15^ These need to be emphasised to interpreters. Interpreting service providers should also follow robust recruitment standards to ensure that qualified interpreters are hired. Regarding interpreting service providers, our study found that some commissioners reported difficulties in holding them accountable, with current KPIs seen as too basic, limiting their effectiveness in ensuring service quality. Our study found that different interpreting providers vary in terms of size, scope and operations, and commissioners may need to set pragmatic KPIs to encourage additional providers in the system, increasing the range of companies that could tender contract bids.

 We found some reports that frontline staff lacked awareness about how interpreting services function. Previous research also suggests that healthcare staff lack awareness of interpreting provision, limiting access for patients.^33^ Two systematic reviews also aligned with our findings that telephone and video interpreting were widely used for accessibility, but often failed to meet patient and provider needs, particularly for complex or sensitive consultations.^34,35^

 Telephone interpreting is regarded as providing on-demand services and other advantages,^36-39^ however, technical glitches and drop-outs impede delivery. Interpreting service providers are usually seen as responsible for these technical issues and used as ‘scape goats’ for poor interpreting delivery. Our results suggest that additional factors such as outdated infrastructure and poor wi-fi signals within healthcare facilities might also impact service quality. This was a surprise finding and highlighted the importance of including a broad range of voices in research studies. More work is needed in upgrading healthcare infrastructure to improve service delivery. There was limited discussion about the role of artificial intelligence (AI) tools in interpreting solutions, except the mention that there is variation in healthcare professionals’ willingness to use these solutions in healthcare. Future research should also map out when different models of interpreting are appropriate/recommended (eg, routine vs. emergency care).

 Our findings show that interpreting services in healthcare and language skills in society are seen as important, with evidence in literature on their impacts on healthcare accessibility and health outcomes.^4,5,7,9,40,41^ However, more work is needed on interpreting service use, costs, benefits, consequences on patient safety, as well as future directions of interpreting services and language skills in wider UK society. Several respondents across the various groups interviewed emphasised the need for non-English speakers to learn English. Although a few negative views were reported – perceptions of interpreting seen as an avoidable cost and questions about why people could not speak English – the majority of participants who emphasised the importance of learning English were from the South Asian/ethnic-minority communities themselves. They highlighted that English proficiency could significantly improve individuals’ quality of life by increasing access to better and less stressful employment opportunities, as well as enabling people to advocate more effectively for themselves during consultations and other interactions. Whilst access to care should not be contingent on language competencies, increasing access to English-learning support programmes through regional government schemes may contribute to reducing inequalities and improving life and socio-economic prospects.^9,42,43^

 Overall, our findings underscore the need for improved standardisation, better regulation, dedicated funding, detailed KPIs, stronger contractual mechanisms and improved interpreter pay and working conditions to enhance the quality, consistency, and accessibility of interpreting services in primary care. A multifaceted approach is needed in addressing identified challenges, which will involve potentially immediate fixable solutions to those involving wider structural changes, including the rapidly evolving AI/digital tool landscape.

###  Strengths and Limitations

 This study moves beyond framing language barriers as a standalone issue in accessing primary healthcare, and instead, examines how interconnected organisational policies and practices shape their uptake and use. We engaged a diverse range of participants, including interpreters, interpreting service providers, local commissioners, and national stakeholders (and also patients and frontline staff in the wider project). This captured diverse perspectives, ensured triangulation, highlighted what each group found important, thereby enabling a nuanced understanding of the factors influencing service delivery. Our study included both public and private sector bodies, demonstrating the interconnectedness of various actors in delivery.

 Many high-income countries have growing numbers of people needing language support. Our study provides key data on the design and implementation of interpreting services for non-English speakers within the UK, providing crucial evidence for other high-income settings.

 We did not explore commissioning and delivery of non-spoken languages, such as for the deaf community. Whilst we inquired about these needs in the frontline staff interviews, we did not explore delivery in-depth. Future studies should explore implementation of non-spoken languages, as delivery of these services may have unique challenges and different policy implications.

## Conclusion

 Our study highlights the critical role of clear policy directives and inter-organisational working in providing interpreting services in primary care. Challenges in funding, standardisation, service accountability, and awareness undermine efforts by commissioners, interpreters, interpreting service providers, primary care staff and other stakeholders to provide an equitable and consistent service to patients. Our findings suggest that improved standardisation, regulatory oversight, and commissioning models tailored to local population needs are essential for enhancing service delivery.

## Acknowledgment

 We are grateful to all commissioners, policy-makers, interpreters, interpreting service providers, and national stakeholders who participated in the interviews, and all staff within their organisations who facilitated the interviews. We would like to thank the INTERPRET-X study Steering Group and Advisory Group for their valuable feedback and insights provided throughout the study.

## Disclosure of artificial intelligence (AI) use

 Not applicable.

## Ethical issues

 The materials and protocol were approved by the University Ethics Committee, University of Surrey (Reference: FHMS-19–20-088) and the Health Research Authority (HRA) and Health and Care Research Wales (HCRW) (REC reference: 23/NE/0066). Relevant documentation was completed for case study site participation in the wider study. Information sheets and written informed consent forms were given and participants who consented took part in the study.

## Conflicts of interest

 Authors declare that they have no conflicts of interest.

## Availability of data and materials

 The datasets used and/or analysed during the current study are not publicly available given the qualitative design and risks to anonymity, but are available from the corresponding authors on reasonable request.

## Supplementary files


Supplementary file 1. Overview of Interview Respondents (n = 31).

Supplementary file 2. Topic Guides for Different Stakeholder Groups.
