# Supplementary material for: Commissioning, Implementation and Policy Perspectives on Interpreting Services in UK Primary Care: A Qualitative Study
Source: Int J Health Policy Manag. 2026 Jul 13;15:9181. doi: 10.34172/ijhpm.9181 (PMC13770220; doi:10.34172/ijhpm.9181)
Supplement: Supplementary file 1 [file ijhpm-15-9181-s001.pdf]

Article title: Commissioning, Implementation and Policy Perspectives on Interpreting Services in UK Primary Care: A Qualitative Study

Journal name: International Journal of Health Policy and Management (IJHPM)

Authors' information: Judith Yargawa[1]*, Katriina L. Whitaker[2], Graham Hieke[2], Cecilia Vindrola-Padros[3], Emily D. Williams[4], Paramjit Gill[5], Lily Islam[6], Sabine Braun[7], Georgia B. Black[1]*

[1]Wolfson Institute of Population Health, Queen Mary University of London, London, UK.

[2]School of Health Sciences, University of Surrey, Guildford, UK.

[3]Department of Targeted Intervention, University College London, London, UK.

[4]Faculty of Life Sciences and Medicine, King's College London, London, UK.

[5]Warwick Applied Health, Warwick Medical School, University of Warwick, Warwick, UK.

[6]Patient and Public Involvement, London, UK.

[7]Centre for Translation Studies, University of Surrey, Guildford, UK.

***Correspondence to:** Judith Yargawa; Email: j.yargawa@qmul.ac.uk & Georgia B. Black Email: g.black@qmul.ac.uk

Citation: Yargawa J, Whitaker KL, Hieke G, et al. Commissioning, implementation and policy perspectives on interpreting services in UK primary care: a qualitative study. Int J Health Policy Manag. 2026;15:9181. doi:10.34172/ijhpm.9181

Supplementary file 1. Overview of Interview Respondents (n = 31).

Descriptor	Frequency (%)
**Role**	
National policymakers	
Healthcare civil servants	7 (22.6)
National think tanks and charities	2 (6.5)
Interpreting regulator and professional interpreting/ translation associations	3 (9.7)
Integrated Care Board commissioners	6 (19.4)
National and local interpreting service providers	6 (19.4)
Interpreters	7 (22.6)
**Gender**	
Male	12 (38.7)
Female	19 (61.3)
**Ethnicity**	
Asian or British Asian	10 (32.3)
Black or Black British	2 (6.5)
White British or White Other	17 (54.8)

Other	2 (6.5)
**Number of years of experience**	
<10 years	14 (45.2)
10-19 years	6 (19.4)
≥20 years	9 (29.0)
Unclear/missing	2 (6.5)
**Number of years in current role**	
<10 years	24 (77.4)
10-19 years	3 (9.7)
≥20 years	2 (6.5)
Unclear/missing	2 (6.5)

* Percentages may not add up to 100% due to rounding-up.
