# Supplementary material for: Commissioning, Implementation and Policy Perspectives on Interpreting Services in UK Primary Care: A Qualitative Study
Source: Int J Health Policy Manag. 2026 Jul 13;15:9181. doi: 10.34172/ijhpm.9181 (PMC13770220; doi:10.34172/ijhpm.9181)
Supplement: Supplementary file 2 [file ijhpm-15-9181-s002.pdf]

Article title: Commissioning, Implementation and Policy Perspectives on Interpreting Services in UK Primary Care: A Qualitative Study

Journal name: International Journal of Health Policy and Management (IJHPM)

Authors' information: Judith Yargawa[1]*, Katriina L. Whitaker[2], Graham Hieke[2], Cecilia Vindrola-Padros[3], Emily D. Williams[4], Paramjit Gill[5], Lily Islam[6], Sabine Braun[7], Georgia B. Black[1]*

[1]Wolfson Institute of Population Health, Queen Mary University of London, London, UK.

[2]School of Health Sciences, University of Surrey, Guildford, UK.

[3]Department of Targeted Intervention, University College London, London, UK.

[4]Faculty of Life Sciences and Medicine, King's College London, London, UK.

[5]Warwick Applied Health, Warwick Medical School, University of Warwick, Warwick, UK.

[6]Patient and Public Involvement, London, UK.

[7]Centre for Translation Studies, University of Surrey, Guildford, UK.

***Correspondence to:** Judith Yargawa; Email: j.yargawa@qmul.ac.uk & Georgia B. Black Email: g.black@qmul.ac.uk

Citation: Yargawa J, Whitaker KL, Hieke G, et al. Commissioning, implementation and policy perspectives on interpreting services in UK primary care: a qualitative study. Int J Health Policy Manag. 2026;15:9181. doi:10.34172/ijhpm.9181

Supplementary file 2. Topic Guides for Different Stakeholder Groups

A. Interview questions for commissioners

Role and overview of experience in commissioning interpreter services

1. I was wondering if we could begin with a description of your current role.

2. Could you give me an overview, in broad terms, of your experiences commissioning interpreter services for patients whose first language is not English?

Selection and process of commissioning interpreter services

I am going to ask a few questions about how you select interpreter services.

1. How are interpreting services commissioned in (*insert ICB/borough group)*? What is the process of commissioning interpreter services in (*insert ICB/borough group)*?
    - How much choice is there with respect to providers/models of interpreting services for South Asian languages?

2. Are there any requirements/eligibility criteria for interpreting service providers before they are commissioned (for example, license, experience, range of languages, etc)?

Funding and payments

I am going to ask a few questions about funding for interpreter services.

1. To what extent is funding a factor in commissioning interpreter services? (*Prompts:* availability of funding, cost of implementation, etc)

2. What funding model is used to pay interpreting service providers (for example, per patient served, fixed contract for block of hours, etc)?

Local population and patients' needs

I am going to ask a few questions about your local population and their needs.

1. What were the local population factors considered in commissioning interpreter services?

2. What impact has interpreting provision had on the health outcomes or other outcomes of your local population?

Perceptions and quality of interpreting services

Now, I am going to ask a few questions about your views on interpreter services.

1. What do you consider a high quality interpreter service (e.g, features etc)?
    - How does this compare to the interpreting service(s) used in your local population?
    - How does the current interpreting service offer address the needs of the South Asian population?

2. Are there any feedback systems in place for assessing quality of the service provided by interpreting providers? *If yes:*

    - What feedback systems are in place? How is the feedback information used?
    - How often are interpreting services reviewed?
    - Are incentives given for quality interpreting provision?

Barriers and facilitators to using interpreter services

We are now getting to the last sets of questions.

1. What would you say are the challenges for patients to accessing and using interpreters? What do you think would encourage patients to use interpreter services?

2. What do you think would make it easier for commissioners to commission or facilitate interpreter services for patients whose first language is not English?

3. *Only ask if this doesn't come up spontaneously from previous questions*: What aspects of the interpreting services for your local population do you think: i) are working well? ii) Not working well?

Closing

- Ask if there’s any other thing participant wants to say on the topic
- Thank participant and close
- Turn off audio-recorder

B. Interview questions for national stakeholders

Role and overview of experience with interpreter services

1. I was wondering if we could begin with a description of your current role.

2. Could you give me an overview of how your role relates to interpreter services?
    - *Prompt on specific parts of participant's response that relate to interpreter services, inequalities, or similar topics*

    - *Possible prompt*: How does inequality fit with the role(s) that you are doing?

Perceptions of interpreting services

I am going to ask a few questions about your views on interpreting services.

1. Could you tell me about your understanding of the current state of interpreter services in the UK?
2. What do you consider a high quality interpreter service (e.g, features etc)?
    a. How does this compare to the current interpreting service provision in the UK, from your experience?

Barriers and facilitators to using interpreter services

I am going to ask a few additional questions.

1. What would you say are the challenges that you have faced in your role or organisation, relating to interpreter services?

2. What do you think would make your job easier around these issues?

3. What do you think would make it easier to provide high quality interpreter services for patients?

4. *Only ask if this doesn't come up spontaneously from previous*: What aspects of the interpreting services in the UK do you think: i) are working well? ii) Not working well?

Closing

- Ask if there's any other thing participant wants to say on the topic
- Thank participant and close
- Turn off audio-recorder

C. Interview questions for interpreters

Staff role and overview of experience in providing interpreter services

1. I was wondering if we could begin with a description of your current role.

2. Could you give me an overview, in broad terms, of your experiences interpreting for patients whose first language is not English?

Involving interpreters

I am going to ask a few questions about how you get involved to interpret.

1. How do you initially get booked to interpret for patients whose first language is not English?

2. How do you get assigned to interpret for patients? What is the process for assigning interpreters to patients?
    - *Prompt*: Are patients able to choose the mode of interpreting they use (i.e., face-to-face, telephone or video)
    - *Prompt*: Can specific interpreters be requested for (e.g., the same interpreter for continuity of care, or an interpreter of the preferred gender)?

Perceptions of interpreting services

Now, I am going to ask a few questions about your views on interpreter services.

1. What do you consider a high quality interpreter service (e.g, features etc)?

2. What's your assessment of how well patients understand the information (diagnosis/treatment options) that you interpret to them regarding their health?

Patients' and interpreters' needs and experiences

1. Are there times where patients may require other types of interpreters as well as for language? For example, sign language for those who are deaf or hard of hearing?

2. Can you talk about times when a patient has arrived but you were unable to interpret?

    a. *Prompt (if info not provided spontaneously)*: Why was it not possible to interpret?
    b. *Prompt*: How did this affect the consultation?
    c. *Prompt*: How was this experience for you as an interpreter?

Barriers and facilitators to using interpreter services

We are now getting to the last sets of questions.

1. What would you say are the challenges for patients to accessing and using interpreters? What are the issues or difficulties regarding access to interpreter services from your perspective?
    - How does the patient's condition/urgency affect this?
    - *Prompt*: Patient reluctance/difficult to arrange/too long for appointments

2. Why do you think patients may be reluctant or turn down interpreter services?

3. What do you think would encourage patients to use interpreter services? What facilitates the access and use of interpreters for patients?

4. What do you think would make it easier for interpreters to interpret/deliver interpreter services to patients whose first language is not English? What facilitates interpreting for interpreters?

Closing

- Ask if there's any other thing participant wants to say on the topic
- Thank participant and close
- Turn off audio-recorder

D. Interview questions for interpreting service providers

Provider role and overview of experience in managing/providing interpreter services

1. I was wondering if we could begin with a description of your current role.

2. Could you give me an overview, in broad terms, of your experiences managing interpreter services for patients whose first language is not English?

Recruitment of interpreters

1. How do interpreters initially come to work for (*insert interpreting service)*? What is the process for becoming an interpreter with your organisation?

2. Are there any requirements (for example, training/qualifications, experience, regional variations/dialects/accents,) for interpreters before they work for (*insert interpreting service)*?

Arranging interpreters

I am going to ask a few questions about how interpreters get assigned to GP practices.

1. How do interpreters get assigned to interpret in GP practices? *Prompts:*
    a. What is the booking process?
    b. When do interpreters interpret face-to-face vs. telephone?
        i. For telephone, how do they get assigned to interpret in 3-way calls?
        ii. How much advanced warning do interpreters get before the consultation?
    c. Can specific interpreters be requested for (e.g., the same interpreter for continuity of care, or an interpreter of the preferred gender)?
    d. How easy or hard is it to get South Asian interpreters?

Perceptions of interpreting services

Now, I am going to ask a few questions about your views on interpreter services.

1. What do you consider a high quality interpreter service (e.g, features etc)?

2. Are there any feedback systems in place for assessing the quality of service provided by interpreters? *If yes:*

    a. What feedback systems are in place? How is the feedback information used?
    b. Are incentives given for quality interpreting provision?

Barriers and facilitators to using interpreter services

We are now getting to the last set of questions.

1. It is not uncommon that sometimes organisations may not be able to provide a service for a variety of reasons. Could you talk about times when a patient has arrived but your service was unable to provide interpreter services?
    a. *Prompt (if info not provided spontaneously)*: Why was it not possible to provide the service?
    b. How was your service able to mitigate this?

2. What would you say are the challenges for patients to accessing and using interpreters? What do you think would encourage patients to use interpreter services?

3. What do you think would make it easier for interpreting services to deliver interpreter services to patients whose first language is not English? What facilitates interpreting for interpreter services?

Closing

- Ask if there's any other thing participant wants to say on the topic
- Thank participant and close
- Turn off audio-recorder
